# Retraction: SATB1 Overexpression Regulates the Development and Progression in Bladder Cancer through EMT

**DOI:** 10.1371/journal.pone.0301572

**Published:** 2024-04-02

**Authors:** The PLOS ONE Editors

This article [1] was identified as one of a group of articles connected by concerns about image reuse [2–13].

The image concerns in this article [1] affect Figs 3 and 6. Specifically,

- In Fig 3, the following western blot results appear similar:
  ○ Fig 3B ZEB2 panel lanes 2–3 and Fig 3B GAPDH panel lanes 1–2 when stretched across the horizontal and vertical axes.
  ○ Fig 3D Snail and Fig 3D Vimentin panels.
- The following panels appear to partially overlap:
  ○ Fig 6A BIU-87 Migration of [1] and Fig 2A MDA-MB-231 miR-181b+CCL18 of [2].
  ○ Fig 6A Control vector Migration of [1] and Fig 2B MCF-7 miR-181b+CCL18 of [2].
  ○ Fig 6A pcDNA3.1-SATB1 Migration of [1] and Fig 2B MCF-7 CCL18 of [2].
  ○ Fig 6A BIU-87 Invasion of [1] and Fig 2A MCF-7 miR-181b+CCL18 of [2].
  ○ Fig 6A Control vector Invasion of [1] and Fig 2A MDA-MB-231 CCL18 of [2].
  ○ Fig 6A pcDNA3.1-SATB1 Invasion of [1], Fig 2A MCF-7 CCL18 of [2], Fig 2B MDA-MB-231 CCL18 of [2], and Fig 2B MDA-MB-231 miR-181b+CCL18 of [2].
  ○ Fig 6B T24 Invasion of [1], Fig 4A Migration Control Vector of [3–4], and Fig 3D Eca-109/pCMV-SOX2-Ctr siRNA of [5].

The authors did not comment on the above concerns.

The *PLOS ONE* Editors retract this article [1] due to the above image concerns that call into question the reliability of the reported results.

FW agreed with the retraction. CC, ZW, XX, HZ, SX, XC, JW, SL, YZ, WX, ZZ, CJ, and ZZ either did not respond directly or could not be reached.
